# Management of Instrument Sterilization Workflow in Endodontics: A Systematic Review and Meta-Analysis

**DOI:** 10.1155/2020/5824369

**Published:** 2020-02-08

**Authors:** Mario Dioguardi, Diego Sovereto, Gaetano Illuzzi, Enrica Laneve, Bruna Raddato, Claudia Arena, Vito Carlo Alberto Caponio, Giorgia Apollonia Caloro, Khrystyna Zhurakivska, Giuseppe Troiano, Lorenzo Lo Muzio

**Affiliations:** ^1^Department of Clinical and Experimental Medicine, University of Foggia, Via Rovelli 50, 71122 Foggia, Italy; ^2^Department of Emergency and Organ Transplantation, Nephrology, Dialysis and Transplantation Unit, University of Bari, Via Piazza Giulio Cesare, 70124 Bari, Italy

## Abstract

Endodontic treatment consists of different working procedures, such as the isolation of the operating field, pulp chamber access, and cleaning and shaping phases with at last the need of a three-dimensional filling of the canals. Each step requires a series of single-use or sterilizable instruments. We have performed a systematic review of different sterilization and disinfection procedures aiming at drawing up a disinfection and sterilization procedure to be used on endodontic instruments. A search on PubMed and Scopus was carried out using the following keywords: “endodontic sterilization,” “endodontic autoclave,” “decontamination dental bur,” “sterilization dental burs,” and “gutta-percha points sterilization.” Eligible articles were included in the qualitative and quantitative analysis. Results of the meta-analysis showed that the most effective method in sterilization is autoclaving. The qualitative analysis showed that the use of single-use or first-use instruments requires presterilization or sterilization procedures, and for reusable tools, attention must be paid to the removal of debris deposited on the blades, not easy to remove manually.

## 1. Introduction

Endodontics is a section of odontology that examines all the pathologies affecting the vital and necrotic dental pulp. It is a discipline that requires the professional to stay concentrated and be accurate for long periods of time in order to perform successfully, and success cannot be separated from the efficiency of the endodontist's instruments [1]. In relation to other dental branches, the use of endodontic instrumentation has important factors to consider: one is the fracture of the intracanal instrumentation, not always possible to eliminate or bypass as such events depend on the torsional and flexural stress during treatment or alterations during the disinfection phase and sterilization [2].

Endodontic instruments vary according to the operational phases and the methods adopted by the operators.

To isolate the operating area rubber dam, rubber dam punch, clamp forceps, dental floss, and spatulas are used, while to open the pulp chamber and remove the carious tissue, diamond burs, multiblades, and burs for removing amalgam and crown are used.

In the glide path and shaping phases, instruments commonly called endodontic files are used, the latter varying depending on the technique adopted by the professional which can be manual or mechanical with constant or variable tapers and with different diameters.

Besides, canal irrigants are used during the shaping phases, such as sodium hypochlorite 5%, EDTA, and chlorhexidine [3] to which the following materials are added to obtain a three-dimensional filling of the canals: gutta-percha or resin cones, zinc oxide-eugenol-based cements, or epoxy resins [4].

The instruments according to their nature can be either disposable or reusable through sterilization processes.

Contamination of the disposable instruments may derive from the production phase if the latter is not supplied as sterile or during dental procedures due to environmental contamination or the patient's biological fluids (saliva, crevicular fluid, blood, and purulent exudate). The oral cavity hosts more than 700 bacterial species that can organize themselves into biofilms and structure themselves into plaque and tartar on the hard surface of the dental elements [5]. It is important that the single-use filling material (gutta-percha or resin cones) does not come into contact with the bacteria commonly present in the oral cavity in order to not contaminate the disinfected and cleansed root canal system with the use of canal irrigants [6].

Reusable instruments can be a source of infection for the professional, and if sterilization and disinfection procedures are not correct, patients may be exposed to an infectious risk too [7]. The instruments used to probe the glide path and shaping come into contact more closely with the patient's deep tissues, such as nerve tissue and vascular tissue; therefore, the risk of cross infection by pathogens is very high [8], and disinfection procedures must necessarily be associated with sterilization procedures. The sterilization methods range from autoclaving at temperatures between 121° and 135°, the use of 2% glutaraldehyde, the use of sterilization glass bead, to dry heat sterilization [9].

The sterilization and disinfection procedures investigated in this research range from the use of autoclaving to glass-bead sterilizations to the use of glutaraldehyde, but also the disinfection and sterilization procedures described in the literature are based on the instrumentation (burs, materials from fillings, and shaping instruments) and disinfectants (hypochlorite, chlorhexidine, and peracetic acid). We have also paid great attention to contaminants such as prions resistant to the procedures commonly used in dentistry.

Endodontics as well as other branches of dentistry comes into close contact with biological fluids and finds itself operating in a field contaminated by the presence of bacteria. These factors mean that the sterilization procedures must be performed well. It is of utmost importance to avoid cross infection [10] and to study and understand the steps of disinfection and sterilization together with the weak points of each step and their influences on the physical and mechanical characteristics of the dental instruments [11]. The aim of this study is also to clarify the disinfection and sterilization procedures in order to propose clear, safe, and practical procedures to the dentists providing oral health services.

In recent years, the studies conducted in this area have focused on the influence of sterilization procedures on endodontic instruments with particular attention to resistance to cyclic and torsional fatigue and to the superficial topographic changes of the instruments. Among the most recent studies comparing sterilization procedures, there is the study by Sheth et al. [9] which compares 2 (autoclaving and glass bed sterilization) of the 3 main sterilization methods that we are going to examine in this systematic review. Another recent study by Kumar et al. [12] investigates the glass-bead sterilization and use of glutaraldehyde.

These two studies, which are the most recent in the endodontic field, have as a result a noncomplete sterilization of the samples analyzed for some methods.

The question we have asked ourselves, in light of these new research studies, is as follows: Which are the methods described in the literature that guarantee the maximum sterility compatibly with the clinical use of endodontic instrumentation?

Through this review, we aim at determining which disinfection and sterilization procedures are the most effective and up-to-date in the endodontic field in order to determine the most efficient procedure to identify instruments no longer suitable to be reused or sterilized.

## 2. Materials and Methods

This review has been conducted based on PRISMA (Preferred Reporting Items for Systematic Reviews and Meta-Analyses) guidelines [13].

Following an initial screening phase, eligible works were included in a quantitative analysis and “outcomes” were judged in order to determine the most efficient methods which determine the complete sterilization of all the studied samples (endodontic instruments).

### 2.1. Eligibility Criteria

The works taken into consideration were peer reviews, original researches, clinical studies (systematic), and reviews related to endodontic sterilization conducted over the last 40 years and published in English. The articles were only selected from the last 40 years because both the sterilization methods and the endodontic instruments have undergone enormous progress: the first with the introduction of increasingly high-performance autoclaves and the second with the introduction of NiTi alloys. In the last 40 years, moreover, the knowledge on infectious diseases previously unrecognized such as AIDS and hepatitis C as well as prion spongiform encephalopathy led to the fact that all the sterilization and disinfection procedures were reviewed and the standards improved.

The articles considered potentially eligible were studies involving disinfection and sterilization:

- Studies were included in the quantitative analysis if they compared methods of sterilization of endodontic instruments and involved a microbiological control analysis.
- Studies were excluded if they did not compare methods of sterilization such as the autoclave, chemical sterilization (glutaraldehyde), and glass-bead sterilizers.

### 2.2. Research Methodology

The studies were identified by using electronic databases and by examining the bibliography in the retrieved articles.

The bibliographic research was conducted on the search engines “PubMed” and “Scopus.” The research on electronic database was conducted between 22 January 2019 and 1 February 2019, and a last search for a partial update of the literature was conducted on 4 February 2019.

The following search terms were entered on PubMed and Scopus: “endodontic sterilization” (PubMed 316 records, Scopus 236 records); “endodontic autoclave” (PubMed 37 records, Scopus 50 records); “decontamination dental burs” (PubMed 11 records, Scopus 6 records); “sterilization dental burs” (PubMed 60 records, Scopus 25 records); “gutta-percha cones sterilization” (PubMed 27 records, Scopus 24 records); and “gutta-percha points sterilization” (PubMed 12 records, Scopus 14 records). Filters for systematic reviews, reviews, and clinical trials were applied to search for terms in order to find previous systematic reviews and to investigate possible outcomes and associative hypotheses that had not been considered yet. For the quantitative analysis, we decided to investigate the comparison of the efficacy between autoclaving sterilization procedures and chemical sterilization (glutaraldehyde) (first outcome) and the comparison between chemical sterilization (glutaraldehyde) and glass-bead sterilizers (second outcome).

### 2.3. Screening Methodology

This research concerns the subsequent screening of the “records” obtained and has been carried out by two independent reviewers; uncertain positions have been discussed with a third reviewer.

The screening included the analysis of the title and the abstract in order to eliminate the records not relevant to the issues of the review; then, we eliminated all the “overlaps.”

The potentially eligible articles were finally submitted to a full-text analysis to verify their use for qualitative analysis; disagreements were solved by a third reviewer, and a fourth reviewer supervised the entire study.

The two reviewers are M. D. and G. I., while the third reviewer is G. T., all dentists from the Department of Clinical and Experimental Medicine of the University of Foggia (Italy). The fourth reviewer, who supervised the project, is L. Lo. M., Director of the Department of Clinical and Experimental Medicine. The K agreement between the 2 screening reviewers was 0.675 (Table 1). It was calculated based on the formulas in the Cochrane Handbook for Systematic Reviews of Interventions (Chapter 7.2.6.1: Calculations for a Simple Kappa Statistic) [14].

The Newcastle–Ottawa scale for case-control studies was used to assess the risk of bias in the included studies [15].

The quantitative analysis was performed with Review Manager software version 5.3 (Cochrane Collaboration, Copenhagen, Denmark).

## 3. Results

A total number of 845 records were identified on the PubMed and Scopus databases (Table 2).

**Figure 1:**
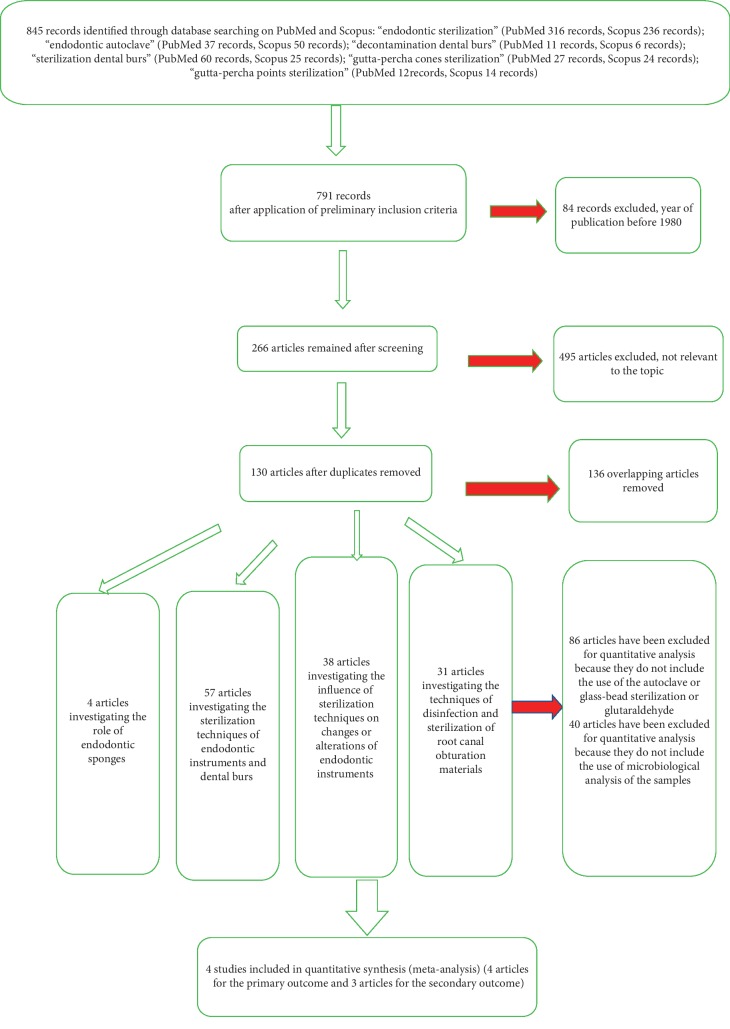
Flow chart of the different phases of the systematic review.

After proceeding with the screening of the articles restricted by the year of publication (last 40 years), we had 761 records. Following the application of the inclusion criteria, at the end of the first screening phase and after the elimination of overlaps, a total of 130 articles were eligible for further analysis. We then decided to highlight the following topics:

1. 57 articles investigating the sterilization procedures of endodontic instruments and burs.
2. 38 articles investigating the influence of sterilization techniques on endodontic instruments.
3. 4 articles investigating the role of endodontic sponges.
4. 31 articles investigating the disinfection and sterilization techniques of root canal filling materials. Aiming at answering to our first and second outcome investigation, a total of four articles were eligible for the quantitative analysis. All selection and screening procedures are described in Table 2 and shown in the flow chart in Figure 1.

### 3.1. Data Extraction

The following studies were included in the quantitative analysis for the first outcome: Kumar et al. [12], Raju et al. [16], Venkatasubramanian et al. [17], and Hurtt and Rossman [18]. The studies selected for the second outcome were by Hurtt and Rossman [18], Raju et al. [16], and Venkatasubramanian et al. [17]. The characteristics of the selected studies are described in Table 3. The data extracted for the two outcomes are described in Tables 4 and 5.

### 3.2. Risk of Bias

The risk of bias was assessed through the Newcastle–Ottawa scale for case-control studies; the results are shown in detail in Table 6. For each category, a value from 1 to 3 was assigned. The study by Morrison and Conrod [19] was excluded from the quantitative analysis because of the risk of bias, being the method of dry heat sterilization similar to glass-bead sterilization. Thus, we did not consider it appropriate to be included in the meta-analysis.

The studies by Raju et al. [16] and Venkatasubramanian et al. [17] have a similar structure, in terms of both the representation of cases and the controls used. They used the same investigation methodology and obtained the same results. Despite the fact that the authors belonged to two different study groups, their studies matched and were perfectly comparable in meta-analysis. Hurt's study [18], despite being the oldest study, used the same methodology of investigation and the same contaminant agent (*Bacillus stearothermophilus*). The endodontic instruments used were the same in these studies, but they were different from those used by Hurtt and Rossman [18] in regard to number and diameter dimensions.

As mentioned above, there is a risk of bias for the Archer Morrisson study [19], as the different method of dry sterilization used for the contaminants is not the same as glass-bead sterilization. In this case, the exposure to bacteria comes from clinical use and oral contamination.

**Figure 2:**
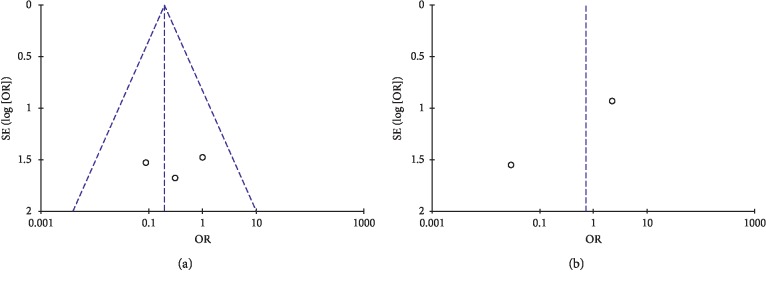
Funnel plot: evaluation of heterogeneity for the first outcome (a) and for the second outcome (b).

The risk of bias and heterogeneity between studies were also assessed with the funnel plot (Figure 2).

### 3.3. Data Analysis

**Figure 3:**
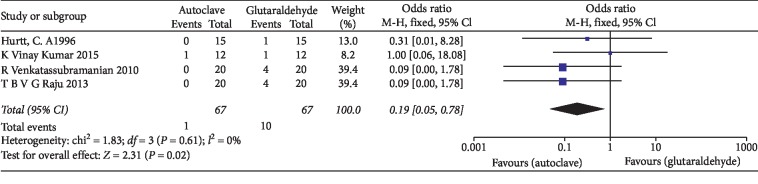
Forest plot of the fixed-effects model of the meta-analysis of the primary outcome.

**Figure 4:**
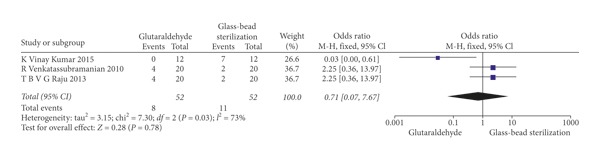
Forest plot of the random-effects model of the meta-analysis of the secondary outcome.

The statistical analysis of data was performed using Review Manager 5.3 software and illustrated using forest plot charts for the *two* outcomes. The comparison of the autoclave and chemical sterilization with glutaraldehyde, meta-analysis, revealed low heterogeneity in the odds ratio, with *I*^2^ values equal to 0%. For such a reason, a fixed-effects model was applied (Figure 3). As for Kumar et al.'s [12] study, two 0 values were shown. When the fixed continuity correction was applied, a value of 1 was added to the boxes containing zero, both for the controls and for the other group. Since the outcome was positive, the results of the forest plots elected the autoclaving method. For the second outcome, the efficacy of using glutaraldehyde was compared to that of using the glass-bead sterilization technique. Since we reported a heterogeneity of *I*^2^ values of 73%, a random-effects model was applied (Figure 4). The results elected chemistry sterilization with glutaraldehyde compared to glass-bead sterilization.

## 4. Discussion of the Meta-Analysis

From the quantitative analysis (meta-analysis) conducted in this review, autoclaving turns out to be the best sterilization method. In fact, all four studies agree on this. For the second outcome, two studies were in favour of glass-bead sterilization and one was not. The statistical analysis reveals that, in all the sterile samples, glutaraldehyde is ahead of glass-bead sterilization. In fact, the forest plot is in favour of the first one. The qualitative analysis of the studies partly confirms what is shown through the quantitative analysis, considering the limited availability of studies for statistical comparison.

The studies examined in this review have shed light on a series of problems faced by practitioners relevant to the sterilization of endodontic instruments.

Concerning the necessity of sterilizing disposable instruments after their first use, we have not obtained any relevant conclusion. Indeed, the lack of sterility due to bacterial contamination and manufacturing residues on the instruments [20] arose in previous studies related to sterilization topics [20]. The need for a phase of decontamination and roughing of the instruments for probing and shaping the canal also must be pointed out; in addition, there is an impossibility to use hot sterilization on all the materials used in endodontics [21], due to physical and mechanical influences on endodontic instruments after the application of detergents, disinfectants, and sterilizing agents [11]. Lastly, prions should be considered for cross infection prevention in both patients and the dental practitioner.

The current authors propose to address the issues listed above by trying to find the most comprehensive and updated answers in the scientific literature. Summarizing, endodontic instruments can undergo the following phases: cleansing and disinfection with removal of the most common residues, the rinsing phase involving drying and packaging, the sterilization phase, and storage of the sterile instruments.

### 4.1. Cleansing and Disinfection (Presterilization)

One of the fundamental phases of the sterilization process is the cleansing and cleaning of coarse debris that is deposited on the endodontic instrumentation. In part, this debris consists of necrotic and protein material, blood residue, and dentinal mud [22].

The methods described in the bibliography involve the use of ultrasonic vessels, disinfectant washers [23], handwashing with immersion in disinfecting liquids/detergents [24], and plasma cleaning [25]. All methods are associated with prewashing in an enzymatic tank with the aim of breaking down the organic components. This prewashing combined with subsequent cleansing and decontamination has the dual effect of reducing the infectious biological risk for the operators and synergistically removing debris [26, 27].

The study conducted by Popovic et al. compares different methods of disinfection and cleansing (immersion in 3% hydrogen peroxide, manual brushing, immersion in 70% alcohol, and then drying; manual brushing, immersion in commercially available disinfectants, rinsing in water, and drying; and manual brushing, soaking in 1% sodium hypochlorite, ultrasonic baths with disinfectants, rinsing in water, and drying) and reports the use of the ultrasonic tray as a method giving efficient results [28].

Other studies which used SEM X-ray analysis [29] showed that both previously unused instruments and brand new ones have some metal residues (nickel chromium) and organic material (carbon) [30]. The authors of the present study recommend the use of the ultrasonic tray to drastically but not completely reduce the amount of residue on these instruments. Eldik et al. also indicated that the use of the ultrasonic tray is essential for the removal of detritus. They focused on the file containers that could, through their design, dampen the sound waves assigned to the removal of residues [31]. In fact, their study showed higher levels of cleanliness on those instruments that were not inside the container carrying tools but were freely immersed in the liquid of the ultrasonic basin. The difference between the two groups was around 5% (80% vs. 85% for tools not inside the containers). In addition, the removal of debris present between the spires of the blades depended on the diameter of the instrument.

As for reducing the contamination of the disposable sterile materials by possible infectious agents, including prions, Smith et al. [32, 33] believed disinfection and sterilization techniques to be ineffective in removing all the debris deposited on the blades of the instruments. They agreed with other authors upon the use of an ultrasound method for the removal of residues to be better than the usage of manual techniques.

The study conducted by Souza et al. in 2011 [33] and that conducted by Smith et al. [32] highlight the importance of using sterile and disposable instrumentation in endodontics. They also highlight how cleansing procedures, such as manual washing, pose a risk for operators. In fact, the authors of these studies recommend the use of ultrasonic trays to achieve greater cleanliness and removal of detritus from endodontic instruments and to reduce the use of aerosol during cleaning and decontamination procedures. The same studies raise the question of how the presence of organic and nonorganic detritus could interfere with the sterilization process by creating a protective layer for bacteria. They provide experimental evidence that such interference does not exist, as the heat of the autoclave is able to destroy all microorganisms [32].

There are valid alternatives to reduce bacterial and viral contamination in the cleansing and decontamination phases, such as the use of washer disinfectors, which have the double effect of breaking down the bacterial load and removing detritus from the blades of endodontic instruments. According to Assaf et al. [34], washer disinfectors are able to remove detritus more effectively than other methods but do not completely remove residue, and their removal rate decreases as the diameter of the endodontic file decreases.

The last method, which is not widely used in the endodontic field but was described by Whittaker et al., is plasma cleaning [25]. This cleansing and disinfection technique involves the use of ionized gases. Plasma cleaning has the advantage of not being aggressive towards the instruments and not releasing substances that are toxic to the workers. The residual gases are usually CO_2_, H_2_O, and N_2_. The present study demonstrates its effectiveness in debris removal (Table 7).

### 4.2. Disinfection of Root Canal Filling Materials

Among the materials used to seal the endodontic root canal system, there are the cones of gutta-percha or resin, in addition to cements based on zinc oxide [35] and eugenol or epoxy resin [36].

The synthesis of the cones occurs under aseptic conditions, but they are subsequently colonized by bacteria and therefore require a preventive use of a system able to decontaminate and sterilize them [37]. The gutta-percha cones contain a certain amount of zinc, which should partly inhibit the growth of microorganisms, but the proliferative action of bacteria occurs anyway.

Contamination of the cones during their production in factories was found in a study by Pang et al. [21], who showed a contamination on 20% of the samples of gutta-percha cones; they were contaminated by bacteria including *Staphylococcus* spp. These data were in agreement with those by Montgomery et al. [38], who reported contamination by bacilli on around 8% of cones, and Gomes et al. [37], who found contamination on 5% of cones (*Staphylococcus epidermidis*).

Bacteria, especially cocci, have the ability to initiate biofilm formation. Moreover, if the root canal filling material is positioned, as often occurring above the apex [39], it could represent a further source of infection for the organism.

Sterilization by heat would alter the cones, so autoclaving sterilization is not suggested.

The methods described in the literature for the disinfection and sterilization of gutta-percha and resin cones are as follows:

1. Sodium hypochlorite in concentrations of 0.5–5% (NaOCl) [22]
2. Chlorhexidine, 2% (CHX) [40]
3. Glutaraldehyde [41]
4. Paraformaldehyde tablets or power [42, 43]
5. Alcohol [44]
6. Peracetic acid, 1-2% [45]
7. Hydrogen peroxide, 3% [46]
8. Polyvinylpyrrolidone-iodine [42, 47, 48]
9. MTAD [49]
10. Saline solution, 0.9% [48]
11. *Rosmarinus officinalis* extract [50]
12. Electron beam accelerator [51]
13. Quaternary ammonium [52]

#### 4.2.1. Sodium Hypochlorite and Chlorhexidine

Sodium hypochlorite has corrosive effects on most endodontic instruments [53]. A study conducted by Valois et al. [54] demonstrated its alterations on gutta-percha cones at concentrations above 2.5%.

Gomes et al. [55] reported superficial alterations on the resin and gutta-percha cones disinfected with chlorhexidine, in contrast with Möller and Örstavik [56], who indicated only a change in the mechanical characteristics with a reduction of the tensile strength. Lee et al. reported a reduction of the tensile strength and elongation of the digester cones immersed in hypochlorite from 1 to 10 days.

A recent study conducted by Grecca et al. [57] has demonstrated, by using an SEM scan, an alteration of the surface of the gutta-percha cones and an alteration partly in the resin ones. This occurs both using 2.5% sodium hypochlorite for 10 min and using 2% chlorhexidine for 15 seconds. These results comply with the study by Gomes et al. [55]. Surface changes are more evident in gutta-percha cones when using 5.25% sodium hypochlorite.

The treatment of gutta-percha or resin with 5% sodium hypochlorite for a contact time of 5 min is one of the most effective methods to reduce the bacterial load. The mechanism of action is related to the oxidative mechanism of the molecule towards the organic component.

In addition to sodium hypochlorite, another very effective decontamination system is the use of 2% chlorhexidine solution. Most of the studies on sterilization methods have demonstrated its effectiveness. In a study by Nabeshima et al. [48], the minimum time taken to obtain optimal results was only one minute vs. 10 min with 1% sodium hypochlorite. The method involved both the cytoplasmic membrane, which induces the discharge of phosphorus and potassium ions, thereby altering the osmotic balance (concentrations between 0.12% and 0.2%), and the cytoplasmic level, through the induction of the precipitation of plasma proteins (concentration 2%)

#### 4.2.2. Glutaraldehyde

Cardoso et al. [58] reported that preparations based on glutaraldehyde were shown to be effective for sterilizing the cones by spore killing after a 15 min exposure. In contrast, Ozalp et al. [41] revealed ineffectiveness for an exposure time equal to 15 minutes, concluding that, to achieve sterility by glutaraldehyde exposure, 8–12 hours are required. The problems with the use of solutions based on glutaraldehyde are both the toxicity of these products and the time required to achieve sterility.

*(1) Peracetic Acid*. Another substance examined and commonly used in the food industry and hospitals as a disinfectant is peracetic acid. This substance is effective against bacteria, fungi, viruses, and spores and requires short time. Unlike most chemical disinfectants, it is not inactivated by the presence of organic materials, leaves no residue, and does not produce by-products that are harmful to the environment. Its mechanism of action involves the release of free oxygen and hydroxyl radicals which decompose into oxygen, water, and acetic acid [45].

A 5 min exposure to 1% peracetic acid showed superior results compared to chlorhexidine and hypochlorite in a study conducted by Subha et al. [45], and according to Salvia et al., when applied at a concentration of 2% for 2.5 min, peracetic acid is able to break down the microbial load almost completely. According to these studies, peracetic acid could be a valid alternative to glutaraldehyde for the disinfection of resin and gutta-percha cones [59].

#### 4.2.3. *Rosmarinus officinalis* Extract

*Rosmarinus officinalis* is a plant of the Lamiaceae family commonly used as an aromatic plant to flavour food. A recent study by Manoel Brito-Júnior et al. [50] has tested the effectiveness of the rosemary extract for the disinfection of cones. Its mechanism of action is probably related to the presence of carnosic acid and carnosol, which may disturb the bacterial cell membrane. Many studies have shown its effectiveness as a bactericidal (gram positive and negative) and a fungicidal agent. Based on this study, it appears that the extract of *Rosmarinus officinalis* can potentially be used in endodontic practice for the disinfection of gutta-percha cones.

#### 4.2.4. Electron Beam Sterilization

Electron beams have the ability to break DNA chains in living organisms, such as bacteria, causing death and rendering the space in which they live sterile. Electron beam treatment is commonly used for the sterilization of medical products and food packaging. A study conducted by Attin et al. [51] demonstrated its effectiveness in reducing the bacterial load on the gutta-percha cones. The effects of beta radiation on gutta-percha polymers, which could modify their internal polymer structure, still need to be investigated.

#### 4.2.5. The Physical Effects of Disinfection and Sterilization Methods on Filling Materials

The alterations on the cones of gutta-percha and resin by the various disinfectants can be summarized in the following points:

- Alterations of the surface due to the action of hypochlorite oxidation on surfaces. Similar alterations are also described for MTAD and chlorhexidine [49].
- The formation of cuboid crystals on the surface by precipitation of hypochlorite with bonds with dissolved components of the polymer of isoprene.
- A reduction of tensile strength following prolonged exposure to the actions of sodium hypochlorite.
- Dimensional variation of the cone, described as elongation.
- All hot methods alter their shape and their mechanical properties.

A study by Maíra Prado et al. recommended the rinsing of the gutta-percha and resin cones after the action of the disinfectant agent in order to remove the chlorine crystals formed on the surface after using sodium hypochlorite [49]. Finally, rinsing with distilled water was strongly recommended after disinfection procedures, especially when NaOCl eMTAD is used. Short et al. [60] recommended, as an alternative to distilled water, 96% ethyl alcohol or 70% isopropyl alcohol. These solutions could cause changes on the surface of the cones and thus compromise the sealing of the filling. Below are reported the majority of the studies examined along with the disinfectants studied in the field of sterilization of filling materials and the conclusions of each scientific work (Table 8).

### 4.3. Sterilization

After a drying and wrapping phase, the sterilization phase is carried out. The most common sterilization techniques used in the last thirty years have been autoclaving, glass-bead sterilization for 45 s at 240°C, UV light at 240–280 nm, laser sterilization, and exposure to glutaraldehyde.

The most commonly used system in endodontics is autoclaving. The authors such as Sheth et al. [9] report that the most efficient system leading to the total destruction of bacteria, viruses, and spores is autoclaving at a minimum temperature of 120°C for 30 min, while the same authors report only on efficacy on endodontic instruments. For sterilization, UV light lamps are used at a frequency of 240–280 nm. The limit of these lamps is that their action is detected only on the surface in direct contact with the light, and inactivation is ineffective for the hollow surfaces of microorganisms. This results from the activation of nucleic acid through the induction of thymine dimers. Also this study reported the use of a glass-bead sterilizer for 45 s at 240°C as ineffective [9].

Another suitable method to sterilize the endodontic instruments and dental instruments in general is the use of 2% glutaraldehyde for 12 h. The effectiveness of this method on dental materials has recently been tested and compared to other methods in a study by Kumar et al. [12]. The authors compared four autoclave methods, glass-bead glutaraldehyde, and a disinfectant detergent based on benzalkonium chloride. According to this study, the ineffective methods for killing microorganisms and spores are the glass bead and the use of the disinfectant (obvious), which both lower the bacterial load but do not sterilize it. The problem with glutaraldehyde certainly is represented by its high toxicity and the time required to obtain a sterilizing effect (12 h), making this method not suitable for practical use in dental surgeries.

The carbon dioxide laser technique is an alternative to sterilization and is rarely used in clinical practice, but according to the studies by Raju et al. [16] and Venkatasubramania et al. [17], it could lead to the sterilization of the endodontic instrumentation to an extent equal to autoclave and superior to chemical sterilization (2-3% glutaraldehyde for 12 h).

Other laser methods were described for the sterilization of reamers in a scientific work by Powell and Whisenant [64] who compared argon, CO_2_, and NdYAG. According to the authors, the method that would guarantee the sterilization of the reamers uses the argon laser.

The use of 6% sodium hypochlorite as a method of sterilization was not shown to be efficient in a recent study performed by Gnau et al. [65]. Countless studies report that heat sterilization (autoclaving for 30 min at 120°C) causes stress on the rotating instrument, which more easily undergoes cyclic fracture during use. In order to have a simple and quick method to sterilize new and performing instruments, the authors proposed the immersion of the new instruments in 6% hypochlorite to reduce the bacterial load. The results shown by the authors agree with previous studies: an exposure time of more than 10 min is able to determine the sterility of the instrument, but a shorter exposure, less than or equal to 5 min, does not guarantee sterility. We should not forget the corrosive effects of sodium hypochlorite on metallic materials and that a prolonged exposure could cause a reduction of the mechanical characteristics of the instrument.

#### 4.3.1. Burs: Decontamination and Sterilization

Among the tools used by the endodontist that are not exempt from cross infection there are the diamond burs, commonly used in the endodontic field for the removal of the carious tissue and the opening of the pulp camera. Studies such as that by Gul et al. [66] report that the diamond cutters supplied by the manufacturers are nonsterile and that the common disinfectant and detritus removal systems such as ultrasonic trays and washer disinfectors are unable to eliminate the detritus collected between the working parts of the drills. Autoclaving is certainly the most effective system for sterilizing the drills [67].

#### 4.3.2. Prions

A very important topic in the field of contaminants is certainly the action by prions, which are responsible for spongiform encephalopathy disease in humans. The prions responsible for the transmission are normally resistant, not denatured, and only partially inactivated by normal disinfection and sterilization procedures.

The risk of cross-contamination of prions in endodontics and more generally in dentistry is low. There are currently no cases of patient-to-patient transmission described in the literature following dental procedures. The risk of such transmission is only theoretical and was described as a hypothesis in the scientific bibliography by Walker et al. [68]. The possibility of transmission comes from the fact that the instruments used to scout and shape the endodontic canal come into contact with organic tissues such as intrapulpal nervous tissue [69]. Studies on prions in people suffering from spongiform encephalopathy have shown that they can be found in the trigeminal nerve, and in theory, this involves the extra-articular and intra-articular nervous tissues [70]. These residues, with the presence of contaminants such as prions, can remain between the blades of the endodontic instruments, resist the presterilization and sterilization procedures, and be inoculated in another patient [68].

The OMS guidelines suggest the following procedures for inactivating prions: immersion in sodium hypochlorite (20,000 ppm of available chlorine) for one hour and heating with 1 M sodium hydroxide for one hour, or autoclaving under vacuum at 121°C for 30–90 min in the presence of sodium hydroxide. These procedures are inappropriate to be used in a dental facility due to the high corrosivity and deterioration of the mechanical properties of the instruments and the timing, not suitable for outpatient activity [71].

### 4.4. Changes in the Physical and Mechanical Properties of Endodontic Instruments Subject to the Sterilization Process

#### 4.4.1. Cyclic Fatigue and Torsional Stress

One of the main causes of fracture of the endodontic instruments used for canal shaping is the cyclic fatigue [72].

The fracture of an instrument is explained by the cyclic stress which the alloy undergoes in a specific section, which involves flexor stress followed by compressive stress. After a number of cycles, the separation of endodontic files occurs [73].

Another cause of fracture is certainly the torsional stress that an instrument subject to rotation inside the channel undergoes when its end is blocked inside the channel [74].

Several studies have investigated the effects of sterility procedures on the mechanical and physical properties of endodontic reamers. Below are the major changes that the instruments undergo following sterilization.

Recently, manufacturers of endodontic instruments have made nickel-titanium alloys which [75], through a thermomechanical production process, produce a superelastic NiTi alloy able to maintain a stable martensitic phase during clinical use [76]. According to Plotino et al. [77], these instruments have the capability to undergo an additional thermal treatment during the sterilization phase which increases their flexibility. Furthermore, other authors, such as Zinelis et al. [78], have suggested that heat treatment during the sterilization phase could reverse the deformations induced by the cyclic fatigue of the instrument during clinical use.

Moreover, according to a study conducted by Alfoqom Alazemi et al. [79], there is a possibility for some nickel-titanium instruments to recover from the shape alteration of the blades occurring during their usage following the sterilization cycle. The deformations were detected under optical magnification, and the numbered instruments were compared after usage.

The studies on the reduction of resistance to cyclic fatigue are conflicting. Most of the studies, mainly the recent ones, report that characteristics of endodontic instruments made with the new NiTi alloy only improve physical characteristics, such as cycle fatigue resistance, during the sterilization phase [80].

In regard to the reduction of torsion resistance following autoclaving, there have also been contrasting studies; some authors (Casper et al. [81]) reported an improvement, especially for newly produced NiTi alloys, other studies had a neutral opinion, supporting a noninfluence on torsional resistance (Hilt et al. [82] and Mize et al. [83]), while others argue that there is an increase of cases of separation of the instrument after hot sterilization due to torsional stress [84].

Previous studies, such as those by Mitchell et al., show that, for steel endodontic instruments, the considerations made for the NiTi alloys are not valid. In fact, there are a reduction in the resistance to torsion and a change in the deflection angle with a reduction in the cutting capability after repeated sterilization cycles [85].

#### 4.4.2. Roughness, Corrosion, and Reduction of Cutting Capability

Five percent sodium hypochlorite (remembering that it is the most used root canal irrigation in endodontics [22]) has corrosive properties on nickel titanium-based alloys [53]. The corrosive action of hypochlorite occurs on the surface of tools, removing a layer of nickel and creating micropitting, which can potentially give rise to cracks that propagate during the cyclical fatigue of the instruments. According to a study by Bulem et al. [86], this corrosion does not affect the mechanical properties of the instruments subject to subsequent sterilization by autoclaving.

A study conducted by Rapisarda et al. [87] claimed that the autoclave induces a surface corrosion condition of the NiTi alloy due to the action of the oxygen that binds the alloy. According to the authors, this corrosive effect produced a reduction in the cutting capacity in 20% of the instruments tested after seven autoclave cycles. Data from a study by Haikenel et al. agreed with this and reported that there is a 1–12% reduction in the cutting capability with 5 to 10 autoclave cycles [88].

A study conducted by Nair et al. [89] using SEM showed that, following sterilization, there is an increase in the roughness of the surface of instruments due to an increase in the irregularities of the surface of the metal alloy that could represent the cores from which the crack starts, and fracture of the instrument occurs under cyclic fatigue [90].

The effects of sterilization procedures on the chemical and physical properties of endodontic instruments can be summarized as follows:

1. Corrosive effects both by disinfecting agents (sodium hypochlorite) through the micropitting phenomenon and by oxygen through bonding and the formation of NiTi oxides under autoclave heat stress [91]
2. Increased surface roughness of the nickel-titanium surface after autoclaving
3. Reduction of cutting capability (NiTi alloys not treated during the hot-making process—twisted file with the M-wire alloy) [92]
4. Partial recovery of macroscopic deformities in NiTi instruments after autoclave sterilization treatment [79]
5. Partial recovery of cyclic fatigue suffered by NiTi instruments (majority but not all studies) in an autoclave [77]
6. Partial recovery of the torsional stress of the major NiTi instruments (but not all studies) in an autoclave [82]
7. Reduction of the cutting angle and the resistance of the steel instruments undergoing autoclaving [88]

### 4.5. Protocol Proposed for the Disinfection of Endodontic Instruments

First of all, instruments should be classified as disposable or new from the factory and instruments that can be reused.

For disposable or new instruments used for shaping, scouting, or glide path, the possibility that they may contain inorganic residues, such as nickel chromium, and carbon residues, should be noted between the spires of the instrument as a result of their working phase, and they may present a certain degree of bacterial contamination such as cocci. Thus, the new or disposable instrumentation must necessarily undergo a phase of detritus removal and decontamination before use. The literature analysis showed that the most suitable procedure for this is the ultrasonic tray or the washer disinfector with the use of nonaggressive disinfectants towards alloys (peracetic acid or quaternary ammonium, excluding hypochlorite at low concentrations due to the phenomenon of micropitting). The instrument must not be put in a container to improve the removal of detritus by ultrasound. Subsequently, the drying and packing phase is carried out. For single-use instruments that are altered by autoclaving and are no longer usable (such as WaveOne Dentsply Maillefer) [93] or steel instruments (K file in steel), if new, the use of autoclaving that is never altered is not recommended (reduction of the capacity for cutting and resistance to cyclic or torsional fatigue), whereas for instruments made with NiTi alloys or new-generation M-wire alloys, the temperature of the autoclave improves the torsional resistance and reduces cyclic fatigue. Alterations, such as an increase in surface roughness, are negligible from a clinical point of view and are not reported in the literature.

The recyclable instruments before being packaged and autoclaved must undergo a decontamination/cleansing phase with the use of enzymatic and proteolytic detergents in order to break down the macroscopic organic residue present on the blades, and at the same time or subsequently, the organic and inorganic residues adhering to the spires of the blades must be removed with ultrasonic or washer disinfector trays. The manual removal of detritus before this phase is strongly advised against for two reasons: to minimize cross infection of the worker and to avoid ineffective manual removal by the operator (even those who are more experienced). We would like to point out that this phase of removal of organic residues is only effective (apart from using single-use instruments) in reducing the risk of transmission of prions in human spongiform encephalopathy. As described above, the prions are only partially denatured by the action of the autoclave.

Following cleansing and decontamination and after the detritus removal phase, the rinsing phase can be carried out with distilled and drying water. After this phase, the operator can handle the decontaminated endodontic material since the bacterial charge is strongly demolished, although not completely eliminated, and in this phase, the assistant's task is to check the macroscopic alterations of the blades of the instruments and to eliminate instruments that are no longer suitable for use (number of times they were used clinically, macroscopic alterations of the blades, and fractured instruments). After this, packaging occurs (always report the sterilization date and the end of sterility, usually one month) as well as autoclaving with cycles at no less than 120°C for 30 min [94]. We would like to remind that NiTi instruments have the capability of recovering partially from autoclaving, but since the physical and mechanical properties only recover partially, we recommend to use the treated instruments for no more than five cycles. To determine how long each type of instrument should be used for, refer to the data provided in the scientific literature and to the indications of use given by the production companies [95].

For the non-autoclavable and disposable material as for gutta-percha or resin cones [96], the most suitable method to reduce the bacterial contamination present on these cones is disinfection by hypochlorite immersion with concentrations ranging from 2% to 5.25% for a minimum time of 5–10 min. Similarly, chlorhexidine can be used in a concentration equal to 2% for a shorter period (data in the literature suggest 1-2 min), or 2% peracetic acid can be used for 5 min [59].

It is important to note that whenever sodium hypochlorite or other acidic detergents are used, the instrument must be rinsed with distilled water or in 90% volume alcohol in order to remove the chlorine crystals that form on the surface of gutta-percha or resin.

We advise against the use of glutaraldehyde as a means to obtain sterility in endodontics, especially for outpatient use. Both the qualitative analysis of the literature and the quantitative analysis showed that the best system for the sterility of endodontic instruments is certainly autoclaving. Remember that glutaraldehyde is a very toxic product for operators, and it requires a very long using time, from 8 to 12 h [97].

## 5. Conclusions

The following considerations emerged from quantitative analyses of the studies and the review of the literature on sterilization and disinfection procedures:

- The quantitative analysis indicated that the most effective method for the sterilization of endodontic instruments is autoclaving.
- Glutaraldehyde sterilization shows more sterile samples than the glass-bead sterilization.
- Disposable or first-use tools require preesterification or sterilization procedures prior to use, both to remove contamination by microorganisms and to remove detritus or residue from the factory.
- The instruments that must be reused after clinical use require careful removal of the processing detritus (organic and inorganic) coming from the patient. The autoclave is partially ineffective for denaturing infectious agents such as prions (even if the subject is at minimal risk).
- The manual removal of detritus on the blades is strongly discouraged due to a higher risk of cross infection.
- Hot sterilization in an autoclave does not alter the mechanical and physical properties of most nickel-titanium instruments.
- For non-autoclavable materials, decontamination through the use of disinfectants (gutta-percha cones) is recommended.

## Figures and Tables

**Table 1:** K agreement calculation.

		Reviewer 2	Reviewer 2	Reviewer 2	Total
		Include	Exclude	Unsure	
Reviewer 1	Include	4	0	0	4
Reviewer 1	Exclude	2	100	4	106
Reviewer 1	Unsure	0	7	13	20
	Total	6	107	17	130

Po = 0.9; Pe = 0.692; K agreement = 0.675 (<0, no agreement; 0.0–0.20, slight agreement; 0.21–0.40, fair agreement; 0.41–0.60, moderate agreement; 0.61–0.80, substantial agreement; 0.81–1.00, almost perfect agreement).

**Table 2:** A complete overview of the search methodology illustrating the keywords used and the number of records obtained for each online database.

Provider search	Keywords	Number of records	Number of records after restriction by year of publication (last 40 years)	Number of remaining articles after screening for the latest review topic	Number of articles investigating the sterilization techniques of endodontic instruments and dental burs	Number of articles investigating the influence of sterilization techniques on changes or alterations of endodontic instruments	Number of articles investigating the role of endodontic sponges	Number of articles investigating the techniques of disinfection and sterilization of root canal obturation materials
PubMed	“endodontic sterilization”	316 records	277 records	71 articles	39 articles	22 articles	4 articles	6 articles
PubMed	“endodontic autoclave”	37 records	36 records	26 articles	18 articles	5 articles	3 articles	0
PubMed	“decontamination dental burs”	11 records	11 records	4 articles	4 articles	0	0	0
PubMed	“sterilization dental burs”	60 records	57 records	22 articles	15 articles	7 articles	0	0
PubMed	“gutta-percha cones sterilization”	27 records	23 records	21 articles	0	0	0	21 articles
PubMed	“gutta-percha points sterilization”	12 records	9 records	7 articles	0	0	0	7 articles
Scopus	“endodontic and sterilization”	263 records	263 records	49 articles	19 articles	20 articles	3 articles	7 articles
Scopus	“endodontic and autoclave”	50 records	50 records	27 articles	6 articles	18 articles	3 articles	0
Scopus	“sterilization and dental burs”	25 records	24 records	13 articles	11 articles	2 articles	0	0
Scopus	“decontamination and dental burs”	6 records	6 records	5 articles	5 articles	0	0	0
Scopus	“gutta-percha and cones and sterilization”	24 records	20 records	15 articles	0	0	0	15 articles
Scopus	“gutta-percha and points and sterilization”	14 records	11 records	6 articles	0	0	0	6 articles
PubMed-Scopus	Number of articles after age restrictions, screening, and subdivision by topic	845 records (no removal of overlaps, no restriction by year of publication)	761 records (number of records after restriction by year of publication, no removal of overlaps, no application of eligibility criteria)	266 records (number of items after application of eligibility criteria)	117 records (number of items after application of eligibility criteria)	74 records (number of items after application of eligibility criteria)	13 records (number of items after application of eligibility criteria)	62 records (number of items after application of eligibility criteria)
Removal of overlaps				130	57	38	4	31

Total articles after screening = 266; after overlaps, 130 were removed.

**Table 3:** Characteristics of the studies potentially eligible for quantitative analysis.

Author	Date	Autoclaving	Carbon dioxide laser sterilization	Chemical sterilization (with glutaraldehyde)	Glass-bead sterilization	Dry hot sterilization	Salt, complete file, 1 min	Salt, complete file, 15 s	Benzalkonium chloride	Salt, blade only	Bacterial contamination	Control	Endodontic instrument diameter and length
Kumar et al. [12]	2015	0/12		0/12	7/12				5/12		Contaminated by oral bacteria during clinical use	12/12	Endodontic files 25 mm
Raju et al. [16]	2013	0/20	0/20	4/20	2/20						*Bacillus stearothermophilus*	20/20	21 mm 25 20 K-files (100)
Venkatasubramanian et al. [17]	2010	0/20	0/20	4/20	2/20						*Bacillus stearothermophilus*	20/20	21 mm 25 20 K-files (100)
Morrison and Conrod[19]	2009	23/40				5/40					Contaminated by oral bacteria during clinical use		Endodontic files 25 mm
Hurtt and Rossman[18]	1996	0/15		1/15			7/15	15/15		15/15	*Bacillus stearothermophilus*	15/15	Hand files (5 of size 10, 5 of size 30, and 5 of size 60)

The study by Archie Morrison was excluded from the statistical analysis.

**Table 4:** Data extracted from the selected studies (primary outcome: autoclaving vs. chemical sterilization).

Author, date, journal	Autoclaving (nonsterile samples)	Chemical sterilization with glutaraldehyde (nonsterile samples)
Kumar et al., 2015, *Journal of International Society of Preventive and Community Dentistry* [12]	0/12	0/12
Raju et al., 2013, *Journal of International Oral Health* [16]	0/20	4/20
Venkatasubramanian R, 2010, *Journal of Indian Society of Pedodontics and Preventive Dentistry* [17]	0/20	4/20
Hurtt and Rossman, 1996, *Journal of Endodontics* [18]	0/15	1/15

**Table 5:** Data extracted from the selected studies (secondary outcome: chemical sterilization vs. glass-bead sterilization).

Author, date, journal	Chemical sterilization with glutaraldehyde (nonsterile samples)	Glass-beadsterilization (nonsterile samples)
Kumar et al., 2015, *Journal of International Society of Preventive and Community Dentistry* [12]	0/12	7/12
Raju et al., 2013, *Journal of International Oral Health* [16]	4/20	2/20
Venkatasubramanian R, 2010, *Journal of Indian Society of Pedodontics and Preventive Dentistry* [17]	4/20	2/20

**Table 6:** Application of the Newcastle–Ottawa scale for case-control studies in order to evaluate the risk of bias of the five studies selected for the present systematic review.

	Selection			Comparability			Exposure		Score
Study	Definition of cases	Representativeness of cases	Selection of controls	Definition of controls	Comparability of cases and controls on the basis of the design or analysis	Ascertainment of exposure	Same method of ascertainment for cases and controls	Nonresponse rate	
Kumar et al. [12]	3	3	3	3	2	3	3	1	21
Raju et al. [16]	3	3	3	3	2	3	3	1	21
Venkatasubramanian et al. [17]	3	3	3	3	2	3	3	1	21
Hurtt and Rossman [18]	3	3	3	3	1	2	3	1	19
Morrison and Conrod [19]	2	2	2	2	2	2	2	1	15 (unsuitable)

**Table 7:** Methods of disinfection and cleaning of sterilizable endodontic instruments with a brief summary of their disadvantages and advantages.

Presterilization methods	Advantages	Disadvantages	Recommended by the scientific literature
Ultrasonic tray	It is the most effective system for debris removal and decontamination	It should be associated with washing with detergent/decontaminating liquids	The majority of the studies report it as the best system for instrument presterilization
Washer disinfector	Effective for decontamination and also debris removal	It should be combined with a prewash	Recommended by the scientific literature immediately after the ultrasound tray
Plasma cleaning	Effective for removing debris, does not release toxic substances, does not induce alterations to the instruments	High cost, little experience in the use in the dental industry	Few studies performed on endodontic instruments, but all agree on its effectiveness
Hand brushing, associated with disinfectants/detergents	Not compared to other methods	Incomplete debris removal, dependent on the operator, risk of cross infection, aggressiveness of disinfectants on the surface of the instruments	Not recommended by the literature

**Table 8:** The majority of the studies examined involving disinfectants studied in the field of sterilization of the filling materials and the conclusions of each scientific work.

First author and date	Journal	Title	Tested disinfectants	Conclusion/results
Grecca, 2011 [57]	*Microscopy Research and Technique*	SEM evaluation of thermoplastic endodontic materials alterations after disinfection: a new experimental model	2.5% NaOCl and 2% CHX	Alteration of the surface of the gutta-percha cones

Ôahinkesen, 2011 [61]	*The Journal of Contemporary Dental Practice*	Evaluation of residual antimicrobial effects and surface changes of gutta-percha disinfected with different solutions	5.25% NaOCl, 2.5% NaOCl, 2% CHX, and 0.05% Octenisept	Exposing gutta-percha to 2% CHX for one minute was found to be the most effective method to eliminate the selected microorganisms

Salvia, 2011 [59]	*Brazilian Oral Research*	Effectiveness of 2% peracetic acid for the disinfection of gutta-percha cones	2% peracetic acid	After a 2.5 min exposure, 100% of the microbial inocula were eliminated

Cleber, 2011 [48]	*Australian Endodontic Journal*	Effectiveness of different chemical agents for disinfection of gutta-percha cones	1% NaOCl, 2% CHX, 10% povidone-iodine, and 0.9% saline solution	2% chlorhexidine gluconate is the most efficient; saline solution is not efficient

Attin, 2001 [51]	*Journal of Endodontics*	Antibacterial properties of electron beam-sterilized gutta-percha cones	Electron beam sterilization	The results of the present study could not demonstrate an influence of electron beam irradiation on the antibacterial properties of the gutta-percha cones

Short, 2003 [60]	*Journal of Endodontics*	The crystallization of sodium hypochlorite on gutta-percha cones after the rapid-sterilization technique: an SEM study	5.25% NaOCl	96% ethyl alcohol, 70% isopropyl alcohol, and distilled water were able to remove chloride crystals that were formed on gutta-percha cones

da Motta, 2001 [62]	*International Endodontic Journal*	Efficacy of chemical sterilization and storage conditions of gutta-percha cones	2.5% NaOCl and 2.2% glutaraldehyde	2.5% sodium hypochlorite and 2.2% glutaraldehyde proved to be effective sterilizing agents for gutta-percha cones, with sodium hypochlorite requiring a shorter period of use

Gomes, 2005 [37]	*Oral Surgery, Oral Medicine, Oral Pathology, Oral Radiology, and Endodontology*	Disinfection of gutta-percha cones with chlorhexidine and sodium hypochlorite	CHX and 5.25% NaOCl	5.25% NaOCl is an effective agent for the rapid disinfection of gutta-percha cones

De Souza, 2003 [42]	*Pesquisa Odontológica Brasileira*	In vitro evaluation of different chemical agents for the decontamination of gutta-percha cones	5.25% NaOCl, 10% polyvinylpyrrolidone-iodine, and paraformaldehyde tablets	Efficient for disinfection

Prado, 2011 [49]	*Oral Surgery, Oral Medicine, Oral Pathology, Oral Radiology, and Endodontology*	The importance of final rinse after disinfection of gutta-percha and Resilon cones	5.25% NaOCl, 2% CHX, and MTAD	Alteration of the surface of the gutta-percha cones after rinsing with distilled water

Valois, 2005 [54]	*Journal of Endodontics*	Structural effects of sodium hypochlorite solutions on gutta-percha cones: atomic force microscopy study	0.5%, 2.5%, or 5.25% NaOCl	Alterations of the topography or elasticity of the gutta-percha cone structure

Roberta Redmersk, 2007	*Brazilian Journal of Microbiology*	Disinfection of gutta-percha cones with chlorhexidine	2% CHX	Decontamination of gutta-percha cones within a 5 min exposure
Gomes, 2007 [55]	*Journal of Endodontics*	Residual effects and surface alterations in disinfected gutta-percha and Resilon cones	2% CHX, 5.25% NaOCl, and saline solution	No alteration of the cone surface

Moller and Orstavik, 1985 [56]	*Journal of Dental Research*	Influence of antiseptic storage solutions on physical properties of endodontic gutta-percha points	70% isopropyl alcohol, 5% chloramine, and 0.5% chlorhexidine	Linear dimensional changes

Pang, 2007 [21]	*Journal of Endodontics*	Effects of short-term chemical disinfection of gutta-percha cones: identification of affected microbes and alterations in surface texture and physical properties	5.25% NaOCl, 2% CHX, and ChloraPrep	Alteration of the surface of the gutta-percha cones and contamination of cones by *Staphylococcus* spp.

Ozalp, 2006 [41]	*Journal of Endodontics*	The rapid sterilization of gutta-percha cones with sodium hypochlorite and glutaraldehyde	2% glutaraldehyde or 2.5% NaOCl	2% glutaraldehyde for 15 min is inefficient; 2.5% sodium hypochlorite is efficient

Higgins, 1986 [43]	*Journal of Endodontics*	The use of paraformaldehyde powder for the sterile storage of gutta-percha cones	Paraformaldehyde power	Inefficient

Montgomery, 1971 [38]	*Oral Surgery, Oral Medicine, Oral Pathology*	Chemical decontamination of gutta-percha cones with polyvinylpyrrolidone-iodine	Polyvinylpyrrolidone-iodine gamma rays	6 min of PVP-I exposure was very effective for decontamination

Royal, 2007 [40]	*Journal of Endodontics*	Comparison of 5.25% sodium hypochlorite, MTAD, and 2% chlorhexidine in the rapid disinfection of polycaprolactone-based root canal filling material	5.25% NaOCl, MTAD, and 2% CHX	The results of this investigation show that 5.25% sodium hypochlorite, MTAD, and 2% chlorhexidine are all effective for the rapid disinfection of gutta-percha and Resilon

Cardoso, 1998 [58]	*Journal of Endodontics*	Rapid sterilization of gutta-percha cones with glutaraldehyde	Glutaraldehyde (Glutaron II, Cidex 28, Glutalabor, Banicide, and Anti-G-Plus)	Sporicidal effect after a 15 min exposure

Namazikhah, 2000 [63]	*Journal of the California Dental Association*	Gutta-percha: a look at the need for sterilization		Gutta-percha is not intentionally contaminated and needs decontamination before use

Subha, 2013 [45]	*Journal of Endodontics*	Efficacy of peracetic acid in rapid disinfection of Resilon and gutta-percha cones compared with sodium hypochlorite, chlorhexidine, and povidone-iodine	1% peracetic acid, 3% NaOCl, 2% CHX, and 10% povidone-iodine	The outcome of this study confirmed the efficacy of 1% peracetic acid and 2% chlorhexidine in the rapid disinfection of both Resilon and gutta-percha

Brito-Junior, 2012 [50]	*Acta Odontologica Latinoamericana*	Antibacterial activity of a plant extract and its potential for disinfecting gutta-percha cones	2.5% NaOCl, 2.0% CHX, and *Rosmarinus officinalis* extract	*Rosmarinus officinalis* extract showed bactericidal effects against *Enterococcus faecalis* and the capacity to disinfect GP cones contaminated by it

Lee, 1988	*Yonsei Medical Journal*	An experimental study of the effect of the various antiseptic storage solutions on physical properties of gutta-percha cone	70% isopropyl alcohol, 5% NaOCl, and 2.5% NaOCl	Alterations of the topography or elasticity of the gutta-percha cone structure

Linke, 1983 [46]	*Oral Surgery, Oral Medicine, Oral Pathology*	Effective surface sterilization of gutta-percha points	3% hydrogen peroxide, 95% ethanol, 4.5% NaOCl, and 17% Zephiran concentrate	Efficient for decontamination
